# Structural Analysis of SARS-CoV-2 ORF8 Protein: Pathogenic and Therapeutic Implications

**DOI:** 10.3389/fgene.2021.693227

**Published:** 2021-09-06

**Authors:** Antonio Valcarcel, Antonio Bensussen, Elena R. Álvarez-Buylla, José Díaz

**Affiliations:** ^1^Laboratorio de Dinámica de Redes Genéticas, Centro de Investigación en Dinámica Celular, Universidad Autónoma del Estado de Morelos, Cuernavaca, Mexico; ^2^Centro de Ciencias de la Complejidad, Universidad Nacional Autónoma de México, Ciudad de México, Mexico; ^3^Laboratorio de Genética Molecular, Epigenética, Desarrollo y Evoluciónde Plantas, Instituto de Ecología, Universidad Nacional Autónoma de México, Ciudad de México, Mexico

**Keywords:** SARS-CoV-2, COVID-19, ORF8, structural biology, COVID-19 therapeutics

## Abstract

Current therapeutic strategies and vaccines against SARS-CoV-2 are mainly focused on the Spike protein despite there are other viral proteins with important roles in COVID-19 pathogenicity. For example, ORF8 restructures vesicular trafficking in the host cell, impacts intracellular immunity through the IFN-I signaling, and growth pathways through the mitogen-activated protein kinases (MAPKs). In this mini-review, we analyze the main structural similarities of ORF8 with immunological molecules such as IL−1, contributing to the immunological deregulation observed in COVID-19. We also propose that the blockage of some effector functions of ORF8 with Rapamycin, such as the mTORC1 activation through MAPKs 40 pathway, with Rapamycin, can be a promising approach to reduce COVID-19 mortality.

## Introduction

The SARS-CoV-2 appeared in Wuhan at the end of December 2019 with the consequent crisis in the health systems due to the lack of an effective treatment to face a then unknown disease with a mortality of 10%. The implementation of physical distancing leads to an overall reduction in incidence by 13% (Islam et al., 2020). At the time of writing, 190 million infections and 4.14 million deaths have been reported. Although the mortality rate is reducing the number of infected patients is increasing, and there is still no effective pharmacological protocol against the disease. More than a year after the start of the pandemic, available vaccines are still uncertain since the virus genome has shown high genetic variability (Islam et al., 2020). Therefore, new drug strategies are needed for the prevention and treatment of the infection caused by this virus and the aftereffects of the disease. One of the most relevant characteristics of the virus is the strong immune response in some patients, in addition to some long-lasting pathologic consequences observed in convalescence patients.

**FIGURE 1:**
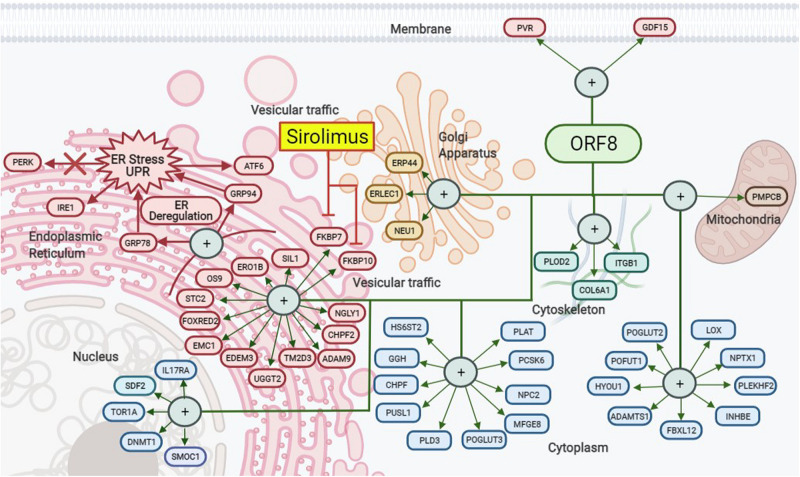
SARS-CoV-2 ORF8 protein interaction map. ORF8 modulates vesicular traffic through the unfolded protein response (UPR) and, therefore, ER stress by stimulating the ATF6 and IRE1 pathways through the upregulation of the GRP78 and GRP94 chaperones. Likewise, this process is linked to other intracellular interactions in the nucleus, Golgi apparatus, cytoskeleton, mitochondria, cytoplasm, and membrane. Rapamycin (Sirolimus) could indirectly decrease the effects of ORF8 by blocking FKBP7 and FKBP10 but not PERK pathway. Created with BioRender.com.

The proteins encoded in the nine open reading frames (ORFs) of SARS-CoV-2 do not appear to be necessary for viral replication. However, they participate in the modulation of the metabolism of the infected host cells, the vesicular trafficking and packing of new viral particles, and the modification of the innate immunity (Gordon et al., 2020). From this group of proteins, ORF8 is the most connected hub with 47 links, and one of these links is the Tor1a (Torsin-1a) protein, that is involved in the quality control of protein folding in the ER (Hill et al., 2018; Gordon et al., 2020). ORF8 acts on ER to modulate the unfolded protein response (UPR) by up regulation of the ER-resident chaperones GRP78 and GRP94 leading to stimulate ATF6 and IRE1 pathways. Although, it does not seem to have any influence on the PERK pathway (Rashid et al., 2021; Figure 1). Thus, during SARS-CoV-2 infection, ORF8 takes the role of a central organizer of the activity of the virus-host hybrid network (the interactome model of viral components with the host proteins) toward the production of new virions (Díaz, 2020).

Coronaviruses show high genetic variability, and the structure of the SARS-CoV-2 genome consist of a set of conserved genes with an exceptionally low or null rate of mutation, together with a set of genes with high rate of variation. For example, of the 11,113 ORF8 sequences analyzed by Pereira (2020), the L84S substitution is the mutation that has been positively selected during the course of the pandemic. In 58 sites with this mutation the change in position 84 from leucine (observed in 85% of the sequences) to serine (observed in 15% of the sequences) stands up (Vilar and Isom, 2021; Zinzula, 2021). In the last group, the gene ORF8 (*ORF8*) has a notable tendency to recombine and undergo deletions that exceed the evolutionary capacity of its analogs in other coronaviruses, facilitating SARS-CoV-2 adaptability to new reservoirs and hosts (Abdelrahman et al., 2020; Zinzula, 2021). Despite the fact that truncations in ORF8 become more common as the pandemic progresses, and that these changes have apparently no influence on the replication of the virus, they are associated with non-synonymous mutations that increases the affinity of protein S for its receptor producing genetic variants with greater contagion capacity and an increased epidemiological persistence (Pereira, 2020).

During the first 6 months of the 2020 pandemic, 240 different non-synonymous mutations and 2 deletions in *ORF8* have been found in 45,400 sequences. Approximately, 50% of these mutations are detrimental to the ORF8 protein, and 25% of them are among the conserved amino acids of other variants of coronavirus in animals. These mutations, regardless of their effects on ORF8 itself, can influence the biology of SARS-CoV-2 and slow down the discovery of new drugs, vaccines, and diagnostics against this coronavirus (Chan et al., 2020; Velazquez-Salinas et al., 2020; Alkhansa et al., 2021). An observational cohort study made in Singapore in the first 3 months of 2020, highlighted that an infection process with the D382 ORF8 variant induced late onset of pneumonia with milder symptoms, compared to the patients infected with the wild type (WT) ORF8. This result was associated with a lower probability of developing hypoxia and a better recovery from the disease (Young et al., 2020), possibly due to an elicited immune response in the absence of a fully functional ORF8. The most distinctive characteristic of severe COVID-19 is the accumulation of high levels of pro-inflammatory cytokines, chemokines, and growth factors that are systemically released and are associated with lung injury. However, in patients infected with the D382 ORF8 variant all these molecules were found in lower concentrations together with high levels of gamma interferon (IFN-γ), and other cytokines responsible for the activation of T cells, in contrast with patients infected with WT (Su et al., 2020). In 2018, a 29-nucleotide deletion in *ORF8* was reported in the SARS-CoV genome, which was acquired during the first stage of person-to-person transmission. These observations point to the fact that these genomic changes relate to the deletion mutations of *ORF8*, given SARS-CoV-2 some advantage in its process of adaptation to humans (Muth et al., 2018).

The new *ORF8* encodes a 121 amino acid secretory protein with 55.4% nucleotide similarity, and 30% protein identity with SARS-CoV counterpart. However, despite this genomic divergence, they share structural similarities as they both present a cavity with adequate electrostatic charges for protein–protein interaction (Neches et al., 2021). Structurally, SARS-CoV-2 ORF8 is a dimer in which each chain is made up of an alpha helix, followed by six-stranded chain β sheet, and an N-terminal hydrophobic signal peptide (1–15 aa of length) that promotes its import into the ER lumen where it can interact with a wide range of host proteins (Gordon et al., 2020; Rashid et al., 2021). This, together with two dimerization interfaces, means that ORF8 has a high possibility of forming unique complexes that can take part in immunological activity. This dimerization is probably an adaptative characteristic absent in homologs from other coronaviruses (Flower et al., 2020).

Among the functions that ORF8 plays in the evasion of the immune system are the activation of IL-17 signaling pathway, and the promotion of the expression of pro-inflammatory factors, supporting the lower intensity and late response to pneumonia caused by the D382 ORF8 variant. Additionally, association of ORF8 deletion variant (D382 variant) with milder disease outcome strongly supports the importance of ORF8 protein as a therapeutic target against SARS-CoV-2 (Sharma et al., 2021). However, the search for direct inhibition drugs of ORF8 is difficult due to the globular structure and high variability of this viral component. Another distinct function of ORF8 protein, different form SARS-CoV 29 nucleotide deleted versions- ORF8a and ORF8b (Pereira, 2021), is the regulation of the amount of MHC-I on the surface of the infected cell through a mechanism of lysosomal degradation dependent on autophagy. This results in dysregulated and deficient antigen presentation, hindering the recognition and elimination of infected cells (Zhang et al., 2020; de Sousa et al., 2020). Recently, computational experiments of homology modeling and molecular coupling suggested that a high expression of ORF8 and the surface glycoprotein may interact with heme porphyrin in the 1-beta chain of hemoglobin, resulting in a significant decrease in gas exchange processes and aggravating hypoxia in patients with severe disease (Liu and Li, 2020). However, these observations are still under investigation because of their clinical implications.

The joint action of ORF8, Nsp1, and Nsp6 results in a significant decrease in the production of IFN-I through different mechanisms to suppress signaling and produce failures and incorrect immune response, which favors the replication and transmission of the virus, to other host cells (Xia et al., 2020). Another example of this synergy is Nsp5, Nsp7, ORF3b, and M that can act together with ORF8 in more than one cell organelle, as in the case of stress-induced to ER (Figure 1; Gordon et al., 2020).

The diverse immune response evasion strategies generating an adaptative advantage for SARS-CoV-2 survival and propagation could be a result of functional mimicry that intensifies the host-pathogen interaction. An example of this functional mimicry comes from *in silico* simulations of ORF8-substrate complexes with F1 and C3b. The results of the coupling suggest that ORF8 can have interactions based on its mimicry with host targets inside and outside of the ER. Even a high extracellular concentration of ORF8 could have unknown interactions with other cell types different from lung alveolar type 2 cells based on possible putative functions conferred by its Ig-like structure. According to the structural alignment of the monomer of SARS-CoV-2 ORF8 (PDB: 7JTL) with SARS-CoV ORF7a (PDB: 1XAK) (Dali Server, Z-score = 4.6, RMSD = 2.4) they share two sets of structural disulfide bonds generating a fold like Ig conformation (Flower et al., 2020). Using the Dali server (Holm, 2020), result in more than one hundred immunoglobulins reporting a Z score higher than 3.9, and RMSD values between 2.7 and 3.6. The most outstanding results are shown in Table 1, in which it is possible to identify their role in mimicking possible host factors.

**TABLE 1:** Highlights of PDB 7JTL comparative studies using dali server.

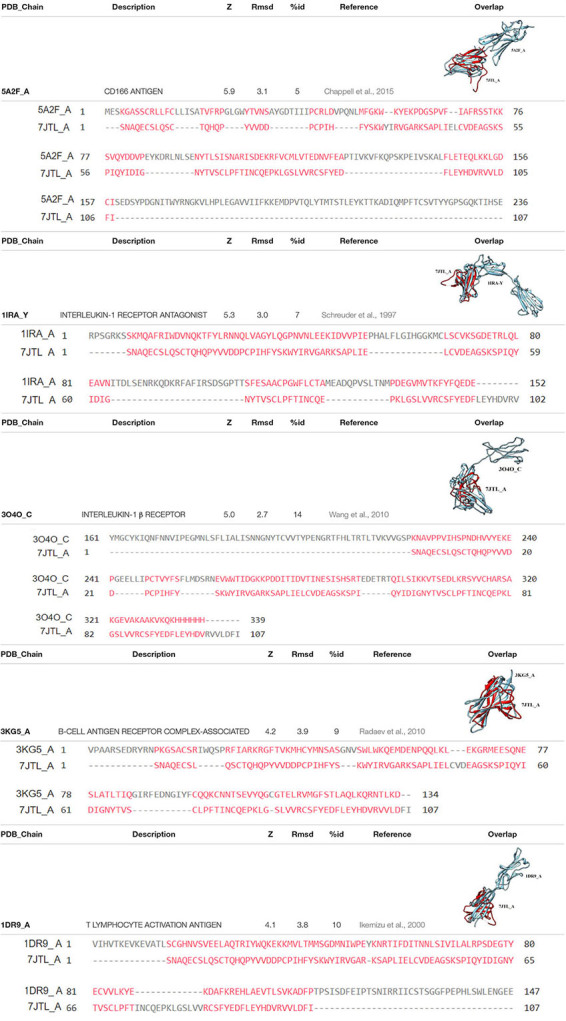

*Match Protein overlap in cyan and ORF8 in red. Alignment conservation setting: 2 Bits.*

ORF8 mimics ALCAM (CD166), which is a structural protein that can activate ERK (Ibáñez et al., 2006). Once activated, ERK stimulates cell growth through the indirect activation of mTORC1 (Saxton and Sabatini, 2017). Some observations *in vitro* from MERS-CoV replication determine that mTORC1 activity is crucial for viral replication, and that the drug Rapamycin can abrogate 60% of the production of new virions (Kindrachuk et al., 2015). Additionally, ORF8 also mimics DNAM-1 (CD266), which is an important molecule that activates Natural Killer (NK) cells (Zhang et al., 2015; Wang et al., 2019), and has been implicated in the regulation of T CD8+ activation (Gilfillan et al., 2008), which can be used by the virus as a potent mechanism to evade the immune response. Moreover, ORF8 also has similarities with OX-2 (CD200) (Hatherley et al., 2013), which is an inhibitory molecule of macrophages (Gordon et al., 2020). Other effect of the structural mimicry of ORF8 is its ability to activate the immune response by itself due to its similarity with the soluble IL-1β receptor and IL-1RA agonists, stimulating the inflammation process. ORF8 also mimics CD79B (3KG5-A) and CD80 (1DR9-A), which are antigens required to activate B and T cell effector functions, respectively (Vasile et al., 1994; Trzupek et al., 2019). However, ORF8 is not precisely equal to such antigens, and can produce an incomplete stimulation of the receptors. In a biological context, incomplete stimulation produces anergy (Rollins and Gibbons, 2017), which may be used by SARS-CoV-2 to enhance its replication.

## Discussion

Current pharmacological strategies to control SARS-CoV-2 infection are mainly focused on inhibiting spike-ACE2 interaction, and to block viral RNA synthesis. Some examples of these drugs are Remdesivir, Lopinavir and Ritonavir, which have been tested on many clinical trials around the world (McKee et al., 2020). Unfortunately, these drugs have little or no effect on patients of COVID-19 (WHO, 2021). Therefore, it is urgent to find novel viral therapeutic targets to control COVID-19. In this regard, evidence shows that cells in which ORF8 is expressed, MHC-I molecules selectively target lysosomal degradation by autophagy and hinder antigen presentation by reducing the recognition and clearance of infected cells. Other pathways of recognition interrupted by the presence of ORF8 are IFN-I signaling, and NF-κB functions. ORF8 also activates the ERK pathway through CD166 signaling (Bouhaddou et al., 2020) and it stimulates growth pathways directly as reported by Gordon et al. (2020). Likewise, ORF8 mimics immune molecules such as IL-1β, activating immunological effector signals from B cells and inhibitory molecules from immune cells such as macrophages, CD8+ T lymphocytes and NK cells (Table 1). These facts pointed out the multi-organizational role of ORF8 inside the host cells. The central question that remains is how all these functions are contributing to ensure viral replication. It seems that all ORF8 interactions are focused on provide an intracellular favorable environment to viral seems that all ORF8 interactions favor a suitable environment for viral replication through activated growth pathways and downregulated immune system, all through the inactivation of macrophages, NK cells, B cells and CD8+ T lymphocytes, making ORF8 a feasible therapeutic target (Supplementary Figure 1). This complex network of interactions contributes to worsen the immune deregulation observed in severe cases of COVID-19 (Pasrija and Naime, 2021).

Consequently, ORF8 is a feasible therapeutic target to simultaneously shut-down viral replication and host immune downregulation. However, *ORF8* is a highly mutating region of the SARS-CoV-2 genome, which decreases the feasibility of ORF8 as a good therapeutic target (Zinzula, 2021), hindering the search for inhibitory drugs. A valid approach to overcome this obstacle is either targeting ORF8 immunological functions or its growth-promoting functions. In general, several RNA viruses such as hepatitis C virus, influenza A virus, Zika virus, and MERS-CoV require a specific metabolic environment and have their own activator mechanisms that ensure the intracellular proliferation of the virus (Karam et al., 2021), In the particular case of SARS-CoV-2, ORF8 can activate the mTOR-PI3K-AKT signaling pathway with a MAPK-dependent process to ensure a proliferative environment. This favorable environment can be blocked with inhibitors of mTORC1 like Rapamycin. It has been reported for the case of MERS-CoV replication that Rapamycin was able to reduce 60% of this virus (Kindrachuk et al., 2015). Thus, the blockage of growth pathways to prevent ORF8 biding interactions can be a better option than the targeting of several immune cells to stop viral infection.

It is possible that Rapamycin, as a co-adjuvant treatment, can improve clinical outcome because it is able to block viral interactions that promotes cell growth, and viral replication. Moreover, Rapamycin can reduce pro-inflammatory cytokines decreasing cell damage in patients with severe COVID-19 (Bischof et al., 2021). The metabolic changes conferred by SARS-CoV-2 infection in renal epithelial cells and lung air-fluid interface (ALI) cultures, showed that SARS-CoV-2 infection reduces the oxidative metabolism of glutamine while maintaining reductive carboxylation, increasing the activity of mTORC1. The work of Mullen et al. (2021) provide evidence of mTORC1 activation in lung tissue from COVID-19 patients, and that mTORC1 inhibitors reduce viral replication in renal epithelial cells and lung ALI cultures. These results suggest that targeting mTORC1 can be a feasible treatment strategy for COVID-19 patients, although more studies are required to determine the mechanism of inhibition and potential efficacy in patients. Rapamycin (Sirolimus) was chosen as it can interact through its methoxy group with the immunophilin binding protein FK506 (FKBP12) forming the rapamycin-FKBP12 complex that is highly specific to the mTOR protein, inhibiting effector processes such as antigen-induced T cell proliferation and cytokine-induced proliferative responses. From the family of polyketide macrolide drugs, Rapamycin (Sirolimus) it is the most studied and unlike Tacrolimus, it does not inhibit calcineurin (PP2B). Despite the fact that the effectiveness of Rapamycin has already been proven as a promising anti-covid drug, the interaction effects with another anti-inflammatory compounds are still to be discovered and open the possibility to have better therapeutic results with lower doses, avoiding toxic effects during the treatment.

The actual evidence shows that the variations observed in the most unstable region of the SARS-CoV-2 genome result in changes in the structure and functions of a set of proteins that counteract the immune response of the host (Supplementary Figure 1). However, SARS-CoV-2 seems not to have a mechanism that allows viral replication under non-permissive conditions (Figure 1). In consequence, the blockage of the activation of cell growth pathway through the inhibition of mTORC1 activity can be a therapeutic strategy that the virus possibly cannot counteract. Nonetheless, more research is necessary to explore the therapeutic use of Rapamycin against the SARS-CoV-2 infection.

## Conclusion

ORF8 is the most linked protein in the virus-host hybrid molecular network formed during the SARS-CoV-2 infection. The structural properties of ORF8 suggest functional mimicry with several immunological molecules such as the IL-1β receptor, resulting in immune system evasion that helps the virus to adapt to new hosts. Additionally, ORF8 restructures the vesicular trafficking in the host cell, and enhances the activity of the growth pathway through the mitogen-activated protein kinases (MAPKs). However, the high mutation rate of *ORF8* decreases its feasibility as a good therapeutic target. In consequence, the blockage of the activation of cell growth pathway through the inhibition of mTORC1 activity with Rapamycin can be a therapeutic strategy that the virus possibly cannot counteract.

## Author Contributions

AV and AB wrote the manuscript and contributed equally to this work. AV, AB, EÁ-B, and JD conceived the study and discussed the content of the review. JD coordinated the study. All authors contributed to the article and approved the submitted version.

## Conflict of Interest

The authors declare that the research was conducted in the absence of any commercial or financial relationships that could be construed as a potential conflict of interest.

## Publisher’s Note

All claims expressed in this article are solely those of the authors and do not necessarily represent those of their affiliated organizations, or those of the publisher, the editors and the reviewers. Any product that may be evaluated in this article, or claim that may be made by its manufacturer, is not guaranteed or endorsed by the publisher.

## References

[B1] Abdelrahman Z. Mengyuan L. Xiaosheng W. (2020). Comparative review of SARS-CoV-2, SARS-CoV, MERS-CoV, and influenza a respiratory viruses. *Front. Immunol.* 11:552909. 10.3389/fimmu.2020.552909 33013925 PMC7516028

[B2] Alkhansa A. Ghayas L. Loubna E. Z. (2021). Mutational analysis of SARS-CoV-2 ORF8 during six months of COVID-19 pandemic. *Gene Rep.* 23:101024. 10.1016/j.genrep.2021.101024 33490718 PMC7813478

[B3] Bischof E. Siow R. C. Zhavoronkov A. Kaeberlein M. (2021). The potential of rapalogs to enhance resilience against SARS-CoV-2 infection and reduce the severity of COVID-19. *Lancet Healthy Longev.* 2 e105–e111. 10.1016/s2666-7568(20)30068-4 33665645 PMC7906698

[B4] Bouhaddou M. Memon D. Meyer B. White K. M. Rezelj V. V. Correa Marrero M. (2020). The global phosphorylation landscape of SARS-CoV-2 infection. *Cell* 182 685–712.e19. 10.1016/j.cell.2020.06.034 32645325 PMC7321036

[B5] Chan A. P. Choi Y. Schork N. J. (2020). Conserved genomic terminals of SARS-COV-2 as co-evolving functional elements and potential therapeutic targets. *BioRxiv* [Preprint]. 10.1101/2020.07.06.190207 33239366 PMC7690956

[B6] Chappell P. E. Garner L. I. Yan J. Metcalfe C. Hatherley D. Johnson S. (2015). Structures of CD6 and its ligand CD166 give insight into their interaction. *Structure* 23 1426–1436. 10.1016/j.str.2015.05.019 26146185 PMC4533223

[B7] de Sousa E. Ligeiro D. Lérias J. R. Zhang C. Agrati C. Osman M. (2020). Mortality in COVID-19 disease patients: correlating the association of major histocompatibility complex (MHC) with severe acute respiratory syndrome 2 (SARS-CoV-2) variants. *Int. J. Infect. Dis.* 98 454–459. 10.1016/j.ijid.2020.07.016 32693089 PMC7368421

[B8] Díaz J. (2020). SARS-CoV-2 molecular network structure. *Front. Physiol*. 11:870. 10.3389/fphys.2020.00870 32754056 PMC7365879

[B9] Flower T. G. Buffalo C. Z. Hooy R. M. Allaire M. Ren X. Hurley J. H. (2020). Structure of SARS-CoV-2 ORF8, a rapidly evolving coronavirus protein implicated in immune evasion. *BioRxiv* [Preprint]. 10.1101/2020.08.27.270637 33361333 PMC7812859

[B10] Gilfillan S. Chan C. J. Cella M. Haynes N. M. Rapaport A. S. Boles K. S. (2008). DNAM-1 promotes activation of cytotoxic lymphocytes by nonprofessional antigen-presenting cells and tumors. *J. Exp. Med.* 205 2965–2973. 10.1084/jem.20081752 19029380 PMC2605240

[B11] Gordon D. E. Jang G. M. Bouhaddou M. Xu J. Obernier K. White K. M. (2020). A SARS-CoV-2 protein interaction map reveals targets for drug repurposing. *Nature* 583 459–468. 10.1038/s41586-020-2286-9 32353859 PMC7431030

[B12] Hatherley D. Lea S. M. Johnson S. Barclay A. N. (2013). Structures of CD200/CD200 receptor family and implications for topology, regulation, and evolution. *Structure* 21 820–832. 10.1016/j.str.2013.03.008 23602662 PMC3664923

[B13] Hill A. Niles B. Cuyegkeng A. Powers T. (2018). Redesigning TOR kinase to explore the structural basis for TORC 1 and TORC 2 assembly. *Biomolecules* 8:36. 10.3390/biom8020036 29865216 PMC6023025

[B14] Holm L. (2020). DALI and the persistence of protein shape. *Prot. Sci*. 29, 128–140. 10.1002/pro.3749 31606894 PMC6933842

[B15] Ibáñez A. Sarrias M. Farnós M. Gimferrer I. Serra-Pagès C. Vives J. (2006). Mitogen-activated protein kinase pathway activation by the CD6 lymphocyte surface receptor. *J. Immunol.* 177 1152–1159. 10.4049/jimmunol.177.2.1152 16818773

[B16] Ikemizu S. Robert J. C. Gilbert R. J. C. Fennelly J. A. Collins A. V. Harlos K. (2000). Structure and dimerization of a soluble form of B7-1. *Immunity* 12 51–60. 10.1016/S1074-7613(00)80158-2 10661405

[B17] Islam M. R. Hoque M. N. Rahman M. S. Rubayet Ul Alam A. S. M. Akther M. Akter Puspo J. (2020). Genome-wide analysis of SARS-CoV-2 virus strains circulating worldwide implicates heterogeneity. *Sci. Rep.* 10 1–9. 10.1038/s41598-020-70812-6 32814791 PMC7438523

[B18] Karam B. S. Morris R. S. Bramante C. T. Puskarich M. Zolfaghari E. J. Lotfi-Emran S. (2021). MTOR inhibition in COVID-19: a commentary and review of efficacy in RNA viruses. *J. Med. Virol.* 93 1843–1846. 10.1002/jmv.26728 33314219 PMC8159020

[B19] Kindrachuk J. Ork B. Hart B. J. Mazur S. Holbrook M. R. Frieman M. B. (2015). Antiviral potential of ERK/MAPK and PI3K/AKT/MTOR signaling modulation for middle east respiratory syndrome coronavirus infection as identified by temporal kinome analysis. *Antimicrob. Agents Chemother.* 59 1088–1099. 10.1128/AAC.03659-14 25487801 PMC4335870

[B20] Liu W. Li H. (2020). COVID-19:attacks the 1-beta chain of hemoglobin and captures the porphyrin to inhibit human heme metabolism. *ChemRxiv* [Preprint]. 10.26434/chemrxiv.11938173.v9

[B21] McKee D. L. Sternberg A. Stange U. Laufer S. Naujokat C. (2020). Candidate drugs against SARS-CoV-2 and COVID-19. *Pharmacol. Res.* 157:104859. 10.1016/j.phrs.2020.104859 32360480 PMC7189851

[B22] Mullen P. J. Garcia G. Purkayastha A. Matulionis N. Schmid E. W. Momcilovic M. (2021). SARS-CoV-2 infection rewires host cell metabolism and is potentially susceptible to MTORC1 inhibition. *Nat. Commun.* 12:1876. 10.1038/s41467-021-22166-4 33767183 PMC7994801

[B23] Muth D. Corman V. M. Roth H. Binger T. Dijkman R. Theresa Gottula l, et al. (2018). Attenuation of replication by a 29 nucleotide deletion in SARS-coronavirus acquired during the early stages of human-to-human transmission. *Sci. Rep.* 8 1–11. 10.1038/s41598-018-33487-8 30310104 PMC6181990

[B24] Neches R. Y. Kyrpides N. C. Ouzounis C. A. (2021). Atypical divergence of SARS-CoV-2 Orf 8 from Orf 7a within the coronavirus lineage suggests potential stealthy viral strategies in immune evasion. *mBio* 12:e03014–20. 10.1128/mBio.03014-20 33468697 PMC7845636

[B25] Pasrija R. Naime M. (2021). The deregulated immune reaction and cytokines release storm (CRS) in COVID-19 disease. *Int. Immunopharmacol.* 90:107225. 10.1016/j.intimp.2020.107225 33302033 PMC7691139

[B26] Pereira F. (2020). Evolutionary dynamics of the SARS-CoV-2 ORF8 accessory gene. Infection, genetics and evolution. *J. Mol. Epidemiol. Evol. Genet. Inf. Dis*. 85:104525. 10.1016/j.meegid.2020.104525 32890763 PMC7467077

[B27] Pereira F. (2021). SARS-CoV-2 variants combining spike mutations and the absence of ORF8 may be more transmissible and require close monitoring. *Biochem. Biophys. Res. Commun.* 550 8–14. 10.1016/j.bbrc.2021.02.080 33676232 PMC7906533

[B28] Radaev S. Zou Z. Tolar P. Nguyen K. Nguyen A. Krueger P. D. (2010). Structural and functional studies of Igαβ; and its assembly with the B cell antigen receptor. *Structure* 18 934–943. 10.1016/j.str.2010.04.019 20696394 PMC2921123

[B29] Rashid F. Dzakah E. E. Wang H. Tang S. (2021). The ORF8 protein of SARS-CoV-2 induced endoplasmic reticulum stress and mediated immune evasion by antagonizing production of interferon beta. *Virus Res.* 296:198350. 10.1016/j.virusres.2021.198350 33626380 PMC7897408

[B30] Rollins M. R. Gibbons J. R. M. (2017). CD80 expressed by CD8 + T cells contributes to PD-L1-induced apoptosis of activated CD8 + T Cells. *J. Immunol. Res.* 2017:7659462. 10.1155/2017/7659462 29181416 PMC5664331

[B31] Saxton R. A. Sabatini D. M. (2017). MTOR signaling in growth, metabolism, and disease. *Cell* 169 361–371. 10.1016/j.cell.2017.03.035 28388417

[B32] Schreuder H. Tardif C. Trump-Kallmeyer S. Soffientini A. Sarubbi E. Akeson A. (1997). A new cytokine-receptor binding mode revealed by the crystal structure of the IL-1 receptor with an antagonist. *Nature* 386 194–200. 10.1038/386194a0 9062194

[B33] Sharma H. B. Panigrahi S. Sarmah A. K. Dubey B. K. (2021). ORF8 contributes to cytokine storm during SARS-CoV-2 infection by activating IL-17 pathway. *iScience* 24:102293. 10.1016/j.isci.2021.102293 33723527 PMC7942160

[B34] Su Y. C. F. Anderson D. E. Young B. E. Linster M. Zhu F. Jayakumar J. (2020). Discovery and genomic characterization of a 382-nucleotide deletion in ORF7B and Orf8 during the early evolution of SARS-CoV-2. *MBio* 11 1–9. 10.1128/mBio.01610-20 32694143 PMC7374062

[B35] Trzupek D. Dunstan M. Cutler A. J. Lee M. Godfrey L. Jarvis L. (2019). Discovery of CD80 and CD86 as recent activation markers on regulatory T cells by protein-RNA single-cell analysis. *BioRxiv* [Preprint]. 10.1101/706275 PMC7315544 32580776

[B36] Vasile S. Coligan J. E. Yoshida M. Seon B. K. (1994). Isolation and chemical characterization of the human B29 and Mb-1 proteins of the B cell antigen receptor complex. *Mol. Immunol.* 31 419–427. 10.1016/0161-5890(94)90061-2 7514267

[B37] Velazquez-Salinas L. Zarate S. Eberl S. Gladue D. P. Novella I. Borca M. V. (2020). Positive selection of ORF3a and ORF8 genes drives the evolution of SARS-CoV-2 during the 2020 COVID-19 pandemic. *BioRxiv* [Preprint]. 10.1101/2020.04.10.035964 PMC7644918 33193132

[B38] Vilar S. Isom D. G. (2021). One Year of SARS-CoV-2: how much has the virus changed? *Biology* 10:91. 10.3390/biology10020091 33530355 PMC7911924

[B39] Wang D. Zhang S. Li L. Liu X. Mei K. Wang X. (2010). Structural insights into the assembly and activation of IL-1β with its receptors. *Nat. Immunol.* 11 905–911. 10.1038/ni.1925 20802483

[B40] Wang H. Qi J. Zhang S. Li Y. Tan S. Gao G. F. (2019). Binding mode of the side-by-side two-IgV molecule CD226/DNAM-1 to its ligand CD155/Necl-5. *Proc. Natl. Acad. Sci.* 116 988–996. 10.1073/pnas.1815716116 30591568 PMC6338850

[B41] WHO (2021). Repurposed antiviral drugs for Covid-19 — interim WHO solidarity trial results. *N. Engl. J. Med.* 384 497–511. 10.1056/nejmoa2023184 33264556 PMC7727327

[B42] Xia H. Cao Z. Xie X. Zhang X. Chen J. Y. Wang H. (2020). Evasion of type I interferon by SARS-CoV-2. *Cell Rep.* 33:108234. 10.1016/j.celrep.2020.108234 32979938 PMC7501843

[B43] Young B. E. Fong S. W. Chan Y. H. Mak T. M. Ang L. W. Anderson D. E. (2020). Effects of a major deletion in the SARS-CoV-2 genome on the severity of infection and the inflammatory response: an observational cohort study. *Lancet* 396 603–611. 10.1016/S0140-6736(20)31757-8 32822564 PMC7434477

[B44] Zhang Y. Zhang J. Chen Y. Luo B. Yuan Y. Huang F. (2020). The ORF8 protein of SARS-CoV-2 mediates immune evasion through potently downregulating MHC-I. *BioRxiv* [Preprint]. 10.1101/2020.05.24.111823 PMC8201919 34021074

[B45] Zhang Z. Wu N. Lu Y. Davidson D. Colonna M. Veillette A. (2015). DNAM-1 controls NK cell activation via an ITT-like Motif. *J. Exp. Med.* 212 2165–2182. 10.1084/jem.20150792 26552706 PMC4647266

[B46] Zinzula L. (2021). Lost in deletion: the enigmatic ORF8 protein of SARS-CoV-2. *Biochem. Biophys. Res. Commun.* 530 116–124. 10.1016/j.bbrc.2020.10.045 33685621 PMC7577707

