# Supplementary material for: Structural Analysis of SARS-CoV-2 ORF8 Protein: Pathogenic and Therapeutic Implications
Source: Front Genet. 2021 Sep 6;12:693227. doi: 10.3389/fgene.2021.693227 (PMC8450498; doi:10.3389/fgene.2021.693227)
Supplement: Supplementary file 1 [file Data_Sheet_1.PDF]

SARS-CoV-2 ORF8 protein: pathogenic and therapeutic implications

Antonio Valcarcel[1], Antonio Bensussen[1], Elena R. Álvarez-Buylla[2,3*], José Díaz[1*]

[1]Laboratorio de Dinámica de Redes Genéticas, Centro de Investigación en Dinámica Celular, Universidad Autónoma del Estado de Morelos, Cuernavaca, México.

[2]Centro de Ciencias de la Complejidad, Universidad Nacional Autónoma de México, Ciudad de México, México.

3Laboratorio de Genética Molecular, Desarrollo y Evolución de Plantas, Instituto de Ecología, Universidad Nacional Autónoma de México, Ciudad de México, México.

Supplementary Material 1

1 Supplementary Figure

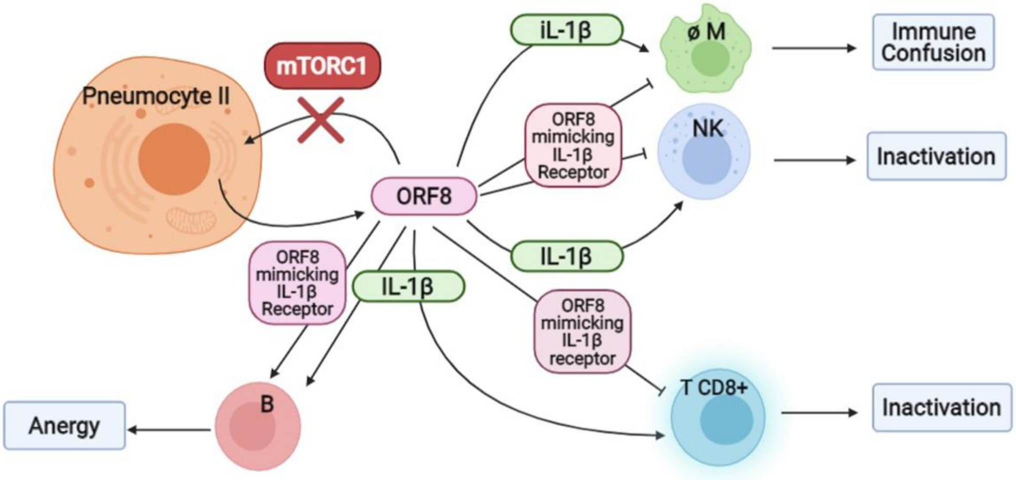

SM Figure 1.- Possible pathogenic mechanism of ORF8 at systemic level. Soluble ORF8 promotes viral replication through indirect stimulation of mTORC1 pathway inside infected cells. Similarly, soluble ORF8 is able to stimulate inflammation by mimicking IL-1β receptor and ligands of IL-1RA. ORF8 can also inhibit NK cells, T CD8+ cells, macrophages and B lymphocytes by mimicking repressor molecules, which in turn can downregulate part of the immune response. Created in BioRender.com.
